# A review of *Copelatus* diving beetles from the Solomon Islands, reporting the discovery of six new species (Coleoptera, Dytiscidae, Copelatinae)

**DOI:** 10.3897/zookeys.1023.61478

**Published:** 2021-03-10

**Authors:** Jiří Hájek, Helena Shaverdo, Lars Hendrich, Michael Balke

**Affiliations:** 1 Department of Entomology, National Museum, Cirkusová 1740, CZ-193 00, Prague 9 – Horní Počernice, Czech Republic National Museum Prague Czech Republic; 2 Naturhistorisches Museum Wien, Burgring 7, 1010, Vienna, Austria Naturhistorisches Museum Wien Vienna Austria; 3 SNSB-Zoologische Staatssammlung München, Münchhausenstraße 21, D-81247, Munich, Germany Zoologische Staatssammlung München Munich Germany; 4 GeoBioCenter, Ludwig-Maximilians-University, Munich, Germany Ludwig-Maximilians-University Munich Germany

**Keywords:** Australian Region, new records, new species, Solomon Islands Archipelago, Taxonomy

## Abstract

The first account of the genus *Copelatus* Erichson, 1832 in the Solomon Islands is provided, reporting 10 species for the Archipelago. Six of these are new to science: *C. baranensis***sp. nov.**, *C. laevipennis***sp. nov.**, *C. urceolus***sp. nov.**, and *C. variistriatus***sp. nov.** from Guadalcanal and *C. bougainvillensis***sp. nov.**, and *C. kietensis***sp. nov.** from Bougainville. *Copelatus tulagicus* Guignot, 1942, described from Tulaghi Island of the Solomons, is recorded from Guadalcanal and Santa Isabel for the first time. The widely distributed Australasian *C. portior* Guignot, 1956 is reported from the Solomon Islands (Guadalcanal and Ontong Java Atoll) for the first time. Two species from Guadalcanal remain unidentified since they are so far known only from a limited number of females.

## Introduction

The circumtropical genus *Copelatus* Erichson, 1832 (Copelatinae) represents the most speciose genus of the family Dytiscidae, with more than 450 described species to date (Nilsson and Hájek 2020). *Copelatus* species inhabit a large variety of both running and stagnant waters, mainly in forested areas of the tropics, and have also been recorded in South America from water tanks in bromeliad plants (Balke et al. 2008) and on Madagascar from wet leaf litter on tropical forest floors (Ranarilalatiana and Bergsten 2019). Recently, one troglomorphic species from Brazil was described from a cave (Caetano et al. 2013).

Most species of *Copelatus* are characterised by longitudinal elytral striae, the number of which has been used to assemble species groups (Sharp 1882; Guignot 1961; Guéorguiev 1968), although this character does not always delineate monophyletic units (Balke et al. 2004; Hájek et al. 2010, 2018). In fact, elytral striation can vary within species and even within sex, and thus the use of this character contributes to confusion in the current classification of *Copelatus* (see, e.g., Hájek et al. 2018; Manuel et al. 2018).

To understand the diversity, distributional patterns, and origin of *Copelatus* in the Australian Region, we have initiated a taxonomic research initiative, starting with the Australian (s. str.) taxa (Hendrich et al. 2019). The present second part addresses the Solomon Islands fauna. Despite of the considerable diversity of *Copelatus* in the Australian Region, only a single species, *Copelatus apicalis* J. Balfour-Browne, 1939 (= *C. tulagicus* Guignot, 1942), has been described from the Solomon Islands to date. However, a short visit by the first author on Guadalcanal, recent collections made by parataxonomists (Kinikoto Mailautoka, Aloysisus Posman) on Guadalcanal and Bougainville respectively, and older material deposited in the Natural History Museum, London, revealed the presence of nine more species in the islands. The material contains six species new to science, which are described here; two other species known only from few female specimens remain unidentified to species level.

## Materials and methods

Exact label data are cited for the type material. Authors’ remarks are found in square brackets: [p] – preceding data are printed, [hw] – preceding data handwritten. Separate label lines are indicated by a slash (/), separate labels by a double slash (//). The specimens included in this study are deposited in the following institutional collections:

**NHMUK**Natural History Museum [former British Museum (Natural History)], London, Great Britain (Christine Taylor);

**LHCM** Lars Hendrich collection, Munich, Germany (property of NHMW);

**NHMW**Naturhistorisches Museum Wien, Vienna, Austria; (Manfred Jäch);

**NMPC**Národní muzeum, Prague, Czech Republic; (Jiří Hájek);

**ZSMG**Zoologische Staatssammlung München, Munich, Germany (part of Staatliche Naturwissenschaftliche Sammlungen Bayern, SNSB) (Michael Balke, Lars Hendrich).

Specimens were examined using an Olympus SZX12 and Leica M205 C stereomicroscopes; measurements were taken with an ocular graticule. Photographs were taken with a Canon EOS 550D digital camera using Canon’s MP-E 65 mm macro lens. Images of the same specimen/structure at different focal planes were combined using Helicon Focus 6.0.3 software. Male genitalia were studied and figured in wet condition. For photography, they were treated with lactic acid; due to that, parts of their apexes less pressed together as in the freshly prepared condition. Aedeagus images were captured by Harald Schillhammer (Vienna, Austria) with a Nikon D4 (in combination with a Novoflex bellows and a Mitutoyo 10/0.25 Apo ELWD) tethered to a PC and controlled with Nikon Camera Control Pro. Resulting image stacks were treated with Zerene Stacker and then post-processed in Adobe Photoshop CS 5.

The species descriptions are provided in the alphabetic order. The descriptive style partly follows Hendrich et al. (2019). The following abbreviations were used in the descriptions:

**TL** total length, measured from clypeal margin to apex of elytra;

**TL-h** total length minus head length, measured from anterior margin of pronotum to apex of elytra;

**MW** maximum width of body measured at right angle to TL.

## Taxonomy

### Copelatus baranensis sp. nov.

Taxon classificationAnimaliaColeopteraDytiscidae

62862C9E-0F9E-5A77-9FF6-92F15E109E59

http://zoobank.org/4A94D800-F6FB-47A6-A534-33E556BE1775

Figures 1 , 15

#### Type locality.

Solomon Islands, Guadalcanal, Barana Village area, Mount Austine, 09°28.0'S, 159°58.4'E.

#### Type material.

***Holotype*:** ♂, labelled: “Solomon Islands, Guadalcanal / Mt. Austine – Barana vill. env. / (secondary forest, gardens, stream) / 09°28.0'S, 159°58.4'E; 280 m / Jiří Hájek leg., 23.xi.–8.xii.2013 [p] // HOLOTYPE ♂ / *COPELATUS* / *baranensis* sp. nov. / Hájek, Shaverdo, Hendrich & Balke det. 2020 [red label, p]” (NMPC).

***Paratypes*:** 1 ♂, 3 ♀♀, same data as holotype (NMPC); 14 ♂♂, 17 ♀♀, labelled: “Solomon Islands, Guadalcanal / ca. 3.5 km SE of Barana vill. / (drying up stream in shaded gorge) / 09°29.8'S, 159°59.5'E; 190 m / Jiří Hájek leg., 24.xi.-14.xii.2013 [p]” (NHMUK, NHMW, NMPC, ZSMG); 6 ♂♂, 6 ♀♀, labelled: “Solomon Islands, Guadalcanal / ca 4.5 km S of Barana vill., forest / nr. “Japanese camp” & Moka river / 09°30.3'S, 159°58.9'E; 275 m / Jiří Hájek leg., 5.–6.xii.2013 [p]” (NMPC); 1 ♀, labelled: “Solomon Islands, Guadalcanal I. / Honiara reg. Barana vill. Env. / 100–300 m XI–XII.2018 / St. Jakl leg.” (LHCM). All paratypes with the respective printed red label.

#### Description of male holotype.

**Figures 1, 2.**
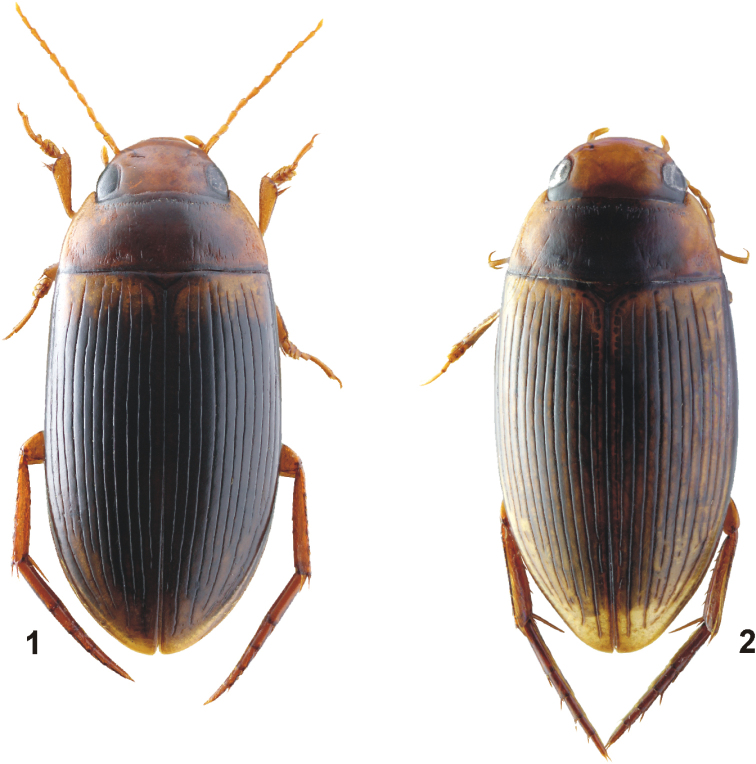
Habitus of *Copelatus***1***C. baranensis* sp. nov. (holotype; TL: 5.9 mm) **2***C. bougainvillensis* sp. nov. (holotype; TL: 5.2 mm).

***Habitus*:** Elongate, oblong-oval, almost parallel-sided, broadest in mid-length of elytra; body moderately convex in lateral view. Body outline continuous, with only indistinct discontinuity between pronotum and elytra. Dorsal surface shiny (Fig. 1).

***Colouration*:** Body colour brown blackish; head, sides of pronotum and appendages paler, ferruginous; base of elytra with irregular transverse testaceous band reaching neither suture nor lateral margin of elytra, band comb-like shaped due to dark colouration of elytral striae; appendages testaceous; ventral part brown blackish.

***Head*:** Moderately broad, ca. 0.64× width of pronotum, trapezoidal. Anterior margin of clypeus indistinctly concave. Antenna with antennomeres long and slender. Reticulation consisting of moderately deeply impressed polygonal isodiametric meshes. Punctation double, consisting of coarse setigerous punctures, and very small punctures spread sparsely on surface; row of coarse punctures present around inner margin of eyes, several punctures present at frontal level of eyes, and anterolaterally to eyes in fronto-clypeal depressions.

***Pronotum*:** Transverse (width/length ratio = 2.54), broadest between posterior angles, lateral margins moderately curved. Sides with lateral beading very thin and indistinct. Reticulation similar to that of head. Punctation similar to that of head; rows of coarse setigerous punctures present along anterior margin, laterally in longitudinal depression close to sides, several punctures present also in basolateral depressions along basal margin. Disc of pronotum laterally with long, irregularly distributed longitudinal strioles; several strioles present also in depressions close to posterior angles. Centre of disc with medial longitudinal smooth line.

***Elytra*:** Base of elytra as broad as pronotal base; lateral margins of elytra subparallel in basal two thirds, curved in apical third. Eleven longitudinal discal striae present on each elytron: stria 1 short, beginning at a fourth of elytral length and ending approximately before apical fifth of elytral length; striae 2 and 3 absent at base; striae 4–6 beginning at base; stria 7 fragmented basally; stria 8 complete; stria 9 absent at base and ending at apical fifth of elytra; stria 10 fragmented into several small strioles, hardly perceptible; stria 11 fragmented apically; all odd striae generally shortened apically. Submarginal stria absent. Surface reticulation similar to that of head and pronotum, meshes slightly smaller and less impressed. Punctation double, consisting of row of coarse setigerous punctures along elytral striae 4, 6, 8 and along lateral margin of elytra, and very fine sparsely distributed punctures.

***Legs*:** Protibia simple, slightly broadened anteriorly, club shaped. Pro- and mesotarsomeres 1–3 distinctly broadened, with adhesive setae on their ventral side.

***Ventral side*:** Prosternum sinuate anteriorly, obtusely keeled medially. Prosternal process shortly lanceolate, in cross-section convex, apex obtuse; process distinctly bordered laterally; reticulation consisting of shallow, hardly perceptible polygonal meshes. Metaventrite with microsculpture consisting of polygonal meshes; lateral parts of metaventrite (“metasternal wings”) tongue-shaped, slender. Metacoxal lines well impressed, nearly complete, absent only close to metaventrite. Metacoxal plates covered with long, deep longitudinal strioles; reticulation consisting of elongated, longitudinal polygonal meshes. Metacoxal processes rounded and indistinctly incised at posterior margin. Abdominal ventrites I and II with longitudinal strioles; ventrites III and IV with oblique strioles laterally. Tuft of setae present antero-medially on ventrites III–V; ventrite VI with setigerous punctures laterally on either side. Abdominal reticulation consisting of elongate polygonal meshes, longitudinal on ventrites I and II, oblique on ventrite III and transverse on ventrites IV–VI. Punctation consisting of fine, sparsely distributed punctures; punctures coarser and denser laterally on apical ventrite.

***Genitalia*:** Median lobe of aedeagus (Fig. 15) sickle-shaped, with obtuse apex and distinct rugose surface sculpture visible in lateral view (Fig. 15A, B); consisting of dorsal and ventral sclerites; dorsal sclerite divided into two parts of different shape in apical half, left part slightly shorter than right one, with lateral margin distinctly curved and apex more or less rounded (Fig. 15C); ventral sclerite with left part strongly sclerotised, its apex in shape of a small, weak hook visible in lateral left view (Fig. 15B), right part membranous; apexes of dorsal and ventral sclerites more or less pressed together.

Lateral lobes (parameres) of narrow triangular form, with almost straight setigerous dorsal margin; setae numerous, dense, and strong distally, and distinctly less numerous, weaker, and sparser basally (Fig. 15D).

**Female.** Identical to male in habitus. Pro- and mesotarsomeres not broadened, without adhesive setae.

#### Variability.

The specimens of the type series vary in extent of the basal testaceous band on elytra. There is also a variation in number and position of longitudinal strioles on pronotum. The highest variability is however, in elytral striation: from complete eleven striae to largely fragmented striae 1, 2, 4, 6, 7, 9, 10; in rare case, stria 1 completely absent; in several specimens, submarginal stria (or several short striae) present at the level of apical fifth of elytral length, sometimes only on one side.

#### Measurements.

TL: 5.4–6.2 mm (mean value: 5.7 ± 0.2 mm); holotype: 5.9 mm. TL-h: 4.9–5.7 mm (mean value: 5.2 ± 0.2 mm); holotype: 5.4 mm. MW: 2.4–2.9 mm (mean value: 2.6 ± 0.1 mm); holotype: 2.6 mm.

#### Differential diagnosis.

Based on the presence of 11 dorsal elytral striae and absence of submarginal stria, the new species can be classified within *C. nigrolineatus* species group. This group contain worldwide only five species: two in Neotropics, one in southern China, one in India, and one species in Australia (Nilsson and Hájek 2020). On the other hand, presence of a submarginal stria in some specimens indicates affiliation of the new species to the *C. trilobatus* species group. The *C. trilobatus* species group includes up to now 24 species occurring in tropics of all continents; two species of the group are occurring in the Papua New Guinea, one species in Fiji, and three species are known from Australia (Nilsson and Hájek 2020).

Based on shape of the male genitalia, *C. baranensis* sp. nov. is not similar to any of the species currently included in the *C. nigrolineatus* group. On the other hand, it is most likely very close to *C. bougainvillensis* sp. nov., *C. kietensis* sp. nov., and to the numerous undescribed species of the *C. trilobatus* species group from New Guinea (H. Shaverdo et al., in preparation). For separation of *C. baranensis* sp. nov., *C. bougainvillensis* sp. nov. and *C. kietensis* sp. nov., see under the two latter species.

#### Etymology.

The new species is named after Barana Village, in the vicinity of which the new species was collected. In this way, the first author would like to thank the people of Barana for their company and help with collecting during the 2013 trip. The specific epithet is an adjective in the nominative singular.

#### Distribution.

The species is known so far only from the small area around Barana Village, south from Honiara City, northern Guadalcanal.

#### Habitat.

At the type locality, the species was collected in small shaded pools of a forest stream (Figs 26, 27). At the other places, the specimens were collected in puddles/pools with muddy bottom made by a temporary forest stream (Fig. 28).

### Copelatus bougainvillensis sp. nov.

Taxon classificationAnimaliaColeopteraDytiscidae

6844BC53-9B11-5F9C-8718-B75ECF77B67D

http://zoobank.org/D01977C9-500E-4752-8DE4-1D9D7652C6C5

Figures 2 , 16

#### Type locality.

Papua New Guinea: Autonomous Region of Bougainville, Kieta.

#### Type material.

***Holotype*:** ♂, labelled: “Papua New Guinea: Bougainville, / Kieta, 620 m, 16.vi.2008, 06.13.035S / 155.30.401E, Posman, (PNG179a) [p] // HOLOTYPE ♂ / *COPELATUS* / *bougainvillensis* sp. nov. / Hájek, Shaverdo, Hendrich & Balke det. 2020 [red label, p]” (ZSMG).

***Paratypes*:** 20 ♂♂, 15 ♀♀, same data as holotype (NHMUK, NHMW, NMPC, ZSMG).

#### Description of male holotype.

***Habitus*:** Elongate, oblong-oval, broadest before mid-length of elytra; body moderately convex in lateral view. Body outline continuous, with only indistinct discontinuity between pronotum and elytra. Dorsal surface shiny (Fig. 2).

***Colouration*:** Body colour ferruginous; head on vertex, disc of pronotum, elytra along striae, and metaventrite darkened, brown blackish.

***Head*:** Moderately broad, ca. 0.64 × width of pronotum, trapezoidal. Anterior margin of clypeus indistinctly concave. Antenna with antennomeres long and slender. Reticulation consisting of moderately deeply impressed polygonal isodiametric meshes. Punctation double, consisting of coarse setigerous punctures, and very small punctures spread sparsely on surface; row of coarse punctures present around inner margin of eyes, several punctures present at frontal level of eyes, and anterolaterally to eyes in fronto-clypeal depressions.

***Pronotum*:** Transverse (width/length ratio = 2.50), broadest between posterior angles; lateral margins almost straight in basal two thirds, slightly curved in anterior third. Sides with lateral beading very thin but distinct, except for anterior angles. Reticulation similar to that of head. Punctation similar to that of head; rows of coarse setigerous punctures present along anterior margin, laterally in longitudinal depression close to sides, several punctures present also in basolateral depressions along basal margin. Disc of pronotum laterally with few short, irregularly distributed longitudinal strioles; several strioles present also in depressions close to posterior angles. Centre of disc with medial longitudinal smooth line.

***Elytra*:** Base of elytra as broad as pronotal base; lateral margins of elytra slightly diverging in basal third, distinctly narrowing in apical half. Eleven discal and a submarginal longitudinal striae present on each elytron: stria 1 beginning posterior from scutellum; striae 2, 4, 6, 8 complete; striae 3, 5, 7, 9, 10 absent at base; stria 9 and 10 somewhat fragmented basally; all odd striae generally shortened apically. Submarginal stria present only as few short striolae in two thirds of elytral length. Surface reticulation similar to that of head and pronotum, meshes slightly smaller and less impressed. Punctation double, consisting of row of coarse setigerous punctures along elytral striae 4, 6, 8 and along lateral margin of elytra, and very fine sparsely distributed punctures.

***Legs*:** Protibia simple, slightly broadened anteriorly, club shaped. Pro- and mesotarsomeres 1–3 distinctly broadened, with adhesive setae on their ventral side. Anterior protarsal claw broad basally, slightly constricted in apical third, strongly curved.

***Ventral side*:** Prosternum sinuate anteriorly, obtusely keeled medially. Prosternal process shortly lanceolate, in cross-section convex, apex obtuse; process distinctly bordered laterally; reticulation consisting of shallow, hardly perceptible polygonal meshes. Metaventrite with microsculpture consisting of polygonal meshes; lateral parts of metaventrite (“metasternal wings”) tongue-shaped, slender. Metacoxal lines well impressed, nearly complete, absent only close to metaventrite. Metacoxal plates covered with long, deep longitudinal strioles; reticulation consisting of elongated, longitudinal polygonal meshes. Metacoxal processes rounded and indistinctly incised at posterior margin. Abdominal ventrites I–II with longitudinal strioles; ventrites III and IV with oblique strioles laterally. Tuft of setae present antero-medially on ventrites III–V; ventrite VI with setigerous punctures laterally on either side. Abdominal reticulation consisting of elongate polygonal meshes, longitudinal on ventrites I and II, oblique on ventrite III and transverse on ventrites IV–VI. Punctation consisting of fine, sparsely distributed punctures; punctures coarser and denser laterally on apical ventrite.

***Genitalia*:** Median lobe of aedeagus (Fig. 16) sickle-shaped, with pointed apex and distinct rugose surface sculpture visible in lateral view (Fig. 16A, B); consisting of dorsal and ventral sclerites; dorsal sclerite divided into two parts of different shape in apical half, left part with lateral margin slightly curved and apex pointed (Fig. 16C); ventral sclerite with left part strongly sclerotised, its apex in shape of a small hook visible in lateral left view (Fig. 16B), right part membranous; apexes of dorsal and ventral sclerites elongate, more or less pressed together.

Lateral lobes (parameres) of narrow triangular form, with almost straight setigerous dorsal margin; setae numerous, dense, and strong distally, and distinctly less numerous, weaker, and sparser basally (Fig. 16D).

**Female.** Identical to male in habitus. Pro- and mesotarsomeres not broadened, without adhesive setae; protarsal claws simple.

#### Variability.

The specimens of the type series vary in extent of infuscation of the pronotal disc and elytra; in some specimens, the pronotum is largely brown blackish with ferruginous sides, and the centre of elytral disc is almost uniformly brown blackish. There is also a variation in number and position of longitudinal strioles on pronotum. Finally, there is a little variability in elytral striation: striae 2–9 may all beginning at the base of elytra, but also the odd striae 3, 5, 7, 9 may be fragmented at the base of elytra.

#### Measurements.

TL: 5.2–5.7 mm (mean value: 5.5 ± 0.1 mm); holotype: 5.2 mm. TL-h: 4.7–5.2 mm (mean value: 5.0 ± 0.1 mm); holotype: 4.7 mm. MW: 2.4–2.7 mm (mean value: 2.6 ± 0.1 mm); holotype: 2.5 mm.

#### Differential diagnosis.

Based on the presence of eleven dorsal striae and a submarginal stria, the new species can be classified within *C. trilobatus* species group, see under *C. baranensis* sp. nov. *Copelatus bougainvillensis* sp. nov. differs from all species of the *C. trilobatus* group by combination of small body length, dorsal surface colouration and the shape of the male genitalia.

On the other hand, in general appearance and structure of the male genitalia, *Copelatus bougainvillensis* sp. nov. is very similar (and probably closely related) to *C. baranensis* sp. nov. It can be distinguished from the latter species by habitus with pronotal sides almost straight in basal two thirds, and elytra diverging in basal third (not subparallel), and by median lobe apically more elongate and more slender, with apex differently shaped (cf. Figs 15, 16). For differentiation from sympatric *C. kietensis* sp. nov., see below.

#### Etymology.

The species is named after the Bougainville Island where it was collected. The specific epithet is an adjective in the nominative singular.

#### Distribution.

The species is to date only known from the type locality on the eastern coast of the Bougainville Island.

#### Habitat.

Unknown.

### Copelatus kietensis sp. nov.

Taxon classificationAnimaliaColeopteraDytiscidae

3DF780DB-DEB9-5077-A231-965FA02566BD

http://zoobank.org/FF97573A-ECD1-4807-9BF5-4A3599B0927B

Figures 3 , 17

#### Type locality.

Papua New Guinea: Autonomous Region of Bougainville, Kieta.

#### Type material.

***Holotype*:** ♂, labelled: “Papua New Guinea: Bougainville, / Kieta, 520 m, 12.vi.2008, 06.12.955S / 155.29.775E, Posman, (PNG180) [p] // HOLOTYPE ♂ / *COPELATUS* / *kietensis* sp. nov. / Hájek, Shaverdo, Hendrich & Balke det. 2020 [red label, p]” (ZSMG).

#### Description of male holotype.

**Figures 3, 4.**
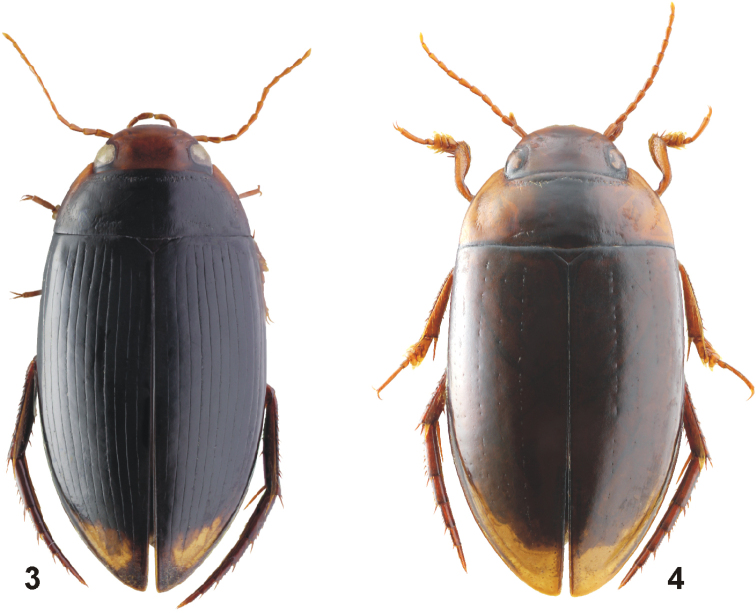
Habitus of *Copelatus***3***C. kietensis* sp. nov. (holotype; TL: 6.3 mm) **4***C. laevipennis* sp. nov. (holotype; TL: 6.9 mm).

***Habitus*:** Elongate, oblong-oval, broadest before mid-length of elytra; body moderately convex in lateral view. Body outline continuous, with only indistinct discontinuity between pronotum and elytra. Dorsal surface shiny (Fig. 3).

***Colouration*:** Head including appendages ferruginous, darkened along eyes; pronotum black with ferruginous anterior angles; scutellar shield ferruginous translucent; elytra black with large irregular subapical testaceous spots; legs ferruginous, hind legs somewhat darker than preceding ones; ventral part of head and prosternum ferruginous, metaventrite, metacoxa and abdominal ventrites darker, blackish brown.

***Head*:** Moderately broad, ca. 0.60 × width of pronotum, trapezoidal. Anterior margin of clypeus indistinctly concave. Antenna with antennomeres long and slender. Reticulation consisting of moderately deeply impressed polygonal isodiametric meshes. Punctation double, consisting of coarse setigerous punctures, and very small punctures spread sparsely on surface; row of coarse punctures present around inner margin of eyes, several punctures present at frontal level of eyes, and anterolaterally to eyes in fronto-clypeal depressions.

***Pronotum*:** Transverse (width/length ratio = 2.79), broadest between posterior angles, lateral margins moderately curved. Sides with lateral beading very thin but distinct, except for anterior angles. Reticulation similar to that of head. Punctation similar to that of head; rows of coarse setigerous punctures present along anterior margin, laterally in longitudinal depression close to sides, several punctures present also in basolateral depressions along basal margin. Disc of pronotum laterally with short, irregularly, and sparsely distributed longitudinal strioles; several strioles present also in depressions close to posterior angles. Centre of disc with superficial medial longitudinal groove.

***Elytra*:** Base of elytra as broad as pronotal base; lateral margins of elytra slightly diverging in basal third, distinctly narrowing in apical half. Eleven discal and a submarginal longitudinal striae present on each elytron: stria 1 shallow, largely fragmented in basal fourth of elytral length and ending approximately before apical fourth of elytral length; striae 2 and 3 shallow, absent at base; striae 4–9 complete; stria 10 absent at base; stria 11 ending at apical fifth of elytra; all odd striae generally shortened apically. Submarginal stria shallow, starting at approximately elytral mid-length. Surface reticulation similar to that of head and pronotum, meshes slightly smaller and less impressed. Punctation double, consisting of row of coarse setigerous punctures along elytral striae 4, 6, 8, and along lateral margin of elytra, and very fine sparsely distributed punctures.

***Legs*:** Protibia simple, slightly broadened anteriorly, club shaped. Pro- and mesotarsomeres 1–3 distinctly broadened, with adhesive setae on their ventral side. Anterior protarsal claw broad basally, strongly curved in apical third.

***Ventral side*:** Prosternum sinuate anteriorly, obtusely keeled medially. Prosternal process shortly lanceolate, in cross-section convex, apex obtuse; process distinctly bordered laterally; reticulation consisting of shallow, hardly perceptible polygonal meshes. Metaventrite with microsculpture consisting of polygonal meshes; lateral parts of metaventrite (“metasternal wings”) tongue-shaped, slender. Metacoxal lines well impressed, nearly complete, absent only close to metaventrite. Metacoxal plates covered with long, deep longitudinal strioles; reticulation consisting of elongated, longitudinal polygonal meshes. Metacoxal processes rounded and indistinctly incised at posterior margin. Abdominal ventrites I and II with longitudinal strioles; ventrites III and IV with oblique strioles laterally. Tuft of setae present antero-medially on ventrites III–V; ventrite VI with setigerous punctures laterally on either side. Abdominal reticulation consisting of elongate polygonal meshes, longitudinal on ventrites I and II, oblique on ventrite III and transverse on ventrites IV–VI. Punctation consisting of fine, sparsely distributed punctures; punctures coarser and denser laterally on apical ventrite.

***Genitalia*:** Median lobe of aedeagus (Fig. 17) sickle-shaped, with pointed apex and distinct rugose surface sculpture visible in lateral view (Fig. 17A, B); consisting of dorsal and ventral sclerites; dorsal sclerite divided into two parts of different shape in apical half, left part with lateral margin slightly curved and apex broadly pointed (Fig. 17C); ventral sclerite with left part strongly sclerotised, its apex in shape of a small hook visible in lateral left view (Fig. 17B), right part less sclerotised; apexes of dorsal and ventral sclerites elongate, more or less pressed together.

Lateral lobes (parameres) of narrow triangular form, with almost straight setigerous dorsal margin; setae numerous, dense, and strong distally, and distinctly less numerous, weaker, and sparser basally (Fig. 17D).

**Female.** Unknown.

#### Measurements.

TL: 6.3 mm. TL-h: 5.7 mm. MW: 3.1 mm.

#### Differential diagnosis.

Based on the presence of 11 dorsal striae + a submarginal stria, the new species can be classified within *C. trilobatus* species group, see under *C. baranensis* sp. nov. *Copelatus kietensis* sp. nov. differs from all species of the *C. trilobatus* group by combination of body length, dorsal surface colouration and the shape of the male genitalia. In particular, *C. kietensis* sp. nov. differs from the sympatric *C. bougainvillensis* sp. nov. in larger body size, broader and more oval habitus, dark colouration of the elytra with testaceous subapical spots, as well as in structure and setation of the male genitalia (cf. Figs 16, 17).

#### Etymology.

The species is named after Kieta, a port town located on the eastern coast of Bougainville Island, where it was collected. The specific epithet is an adjective in the nominative singular.

#### Distribution.

The species is known so far only from the type locality on the eastern coast of the Bougainville Island.

#### Habitat.

Unknown.

### Copelatus laevipennis sp. nov.

Taxon classificationAnimaliaColeopteraDytiscidae

13F48102-D269-5F0F-9E55-656C158A365C

http://zoobank.org/507C2AEA-9E85-4957-A8BC-F3442EC83D7A

Figures 4 , 18 , 23

#### Type locality.

Solomon Islands, Guadalcanal, 0.5 km N of Mbaole Village, 09°37.69'S, 160°06.69'E.

#### Type material.

***Holotype*:** ♂, labelled: “Salomonen: C-Guadalcanal, / 0.5 km N Mbaole, 2799 feet / S 09°37.69 E 160°06.69E / 2007 K. Mailautoka leg. [p] // DNA / M. Balke / 2907 [green label, p] // HOLOTYPE ♂ / *COPELATUS* / *laevipennis* sp. nov. / Hájek, Shaverdo, Hendrich & Balke det. 2020 [red label, p]” (ZSMG).

***Paratypes*:** 1 ♂, same locality data as holotype, with additional label: “DNA / M. Balke / 3334 [green label, p]” (NMPC); 2 ♀♀, labelled: “Solomon Islands: C-Guadalcanal, / 0.5 km N Mbaole, 853 m, 2007, / 09°37.69S, 160°06.88E, K. Mailautoka [p]” (NMPC, ZSMG); 10 ♂♂, 7 ♀♀, labelled: “SOLOMON IS.: / Guadalcanal Is. / Suta / 27.vi.1956 [p] // E.S.Brown / B.M.1957-201 [p]” (NHMUK, NMPC, ZSMG); 1 ♂, labelled: “5327 [on side, hw] / SOLOMON IS. [red underlined] / Guadalcanal [p] / Suta / 27.vi. [hw] 195 [p] 6 [hw] / E.S.Brown [p] / Sangava / R [on reverse, hw] // E.S.BROWN coll / C.I.E. 1957-24 [hw] // E.S.Brown / B.M.1957-201 [p]” (NHMUK); 1 ♂, labelled: “♂ [p] // Type [round label with red frame, p] // SOLOMON IS: / GUADALCANAL / Tenaru R. headwaters. / 1820' 5.viii.53. / In hole in tree trunk. [hw] // Brit.Mus. / 1987-14 [p] // aC 2 [hw] // Copelatus / torosus Type! [hw] / J. Balfour-Browne det., 195 [p] 3 [hw]” (NHMUK). All paratypes with the respective printed red label.

#### Description of male holotype.

***Habitus*:** Elongate, oblong-oval, broadest shortly before mid-length of elytra; body distinctly convex in lateral view. Body outline continuous, without discontinuity between pronotum and elytra. Dorsal surface shiny (Fig. 4).

***Colouration*:** Body colour pitchy brown; sides of pronotum (except for lateral margin) and appendages paler, ferruginous.

***Head*:** Moderately broad, ca. 0.65 × width of pronotum, trapezoidal. Anterior margin of clypeus indistinctly concave. Antenna with antennomeres long and slender. Reticulation consisting of moderately deeply impressed isodiametric meshes. Punctation double, consisting of coarse setigerous punctures, and very small punctures spread sparsely on surface; row of coarse punctures present around inner margin of eyes, few punctures present at frontal level of eyes, and several punctures anterolaterally to eyes in fronto-clypeal depressions.

***Pronotum*:** Transverse (width/length ratio = 2.78), broadest between posterior angles, lateral margins moderately curved. Sides with lateral beading very thin and indistinct. Reticulation similar to that of head. Punctation similar to that of head; rows of coarse setigerous punctures present along anterior margin, laterally in longitudinal depression close to sides, several punctures present also in basolateral depressions along basal margin. Disc of pronotum with shallow medial longitudinal scratch.

***Elytra*:** Base of elytra as broad as pronotal base; lateral margins of elytra subparallel-sided in basal half, distinctly narrowing in apical half. Elytral striae absent. Reticulation similar to that of head and pronotum, meshes somewhat elongated longitudinally. Punctation consisting of coarse setigerous punctures and very fine sparse punctures. Coarse punctures arranged in three distinct longitudinal puncture lines: two discal and one lateral; another row of punctures present along lateral margin of elytra, and few coarse punctures present also in interspace between discal and lateral puncture lines.

***Legs*:** Protibia modified, angled near base, distinctly broadened anteriorly, club shaped. Pro- and mesotarsomeres 1–3 distinctly broadened, with adhesive setae on their ventral side.

***Ventral side*:** Prosternum sinuate anteriorly, obtusely keeled medially. Prosternal process shortly lanceolate, in cross-section convex, apex obtuse; process distinctly bordered laterally; reticulation almost effaced. Metaventrite with microsculpture consisting of polygonal meshes; lateral parts of metaventrite (“metasternal wings”) tongue-shaped, slender. Metacoxal lines nearly complete, absent only very close to metaventrite. Metacoxal plates covered laterally with long, deep longitudinal strioles; reticulation consisting of extremely elongated, longitudinal polygonal meshes. Metacoxal processes rounded and incised at posterior margin. Abdominal ventrites I and II with longitudinal strioles; ventrites III and IV with oblique strioles laterally. Tuft of setae present antero-medially on ventrites III–V; ventrite VI with setigerous punctures laterally on either side. Abdominal reticulation consisting of elongate polygonal meshes, longitudinal on ventrites I and II, oblique on ventrite III and transverse on ventrites IV–VI. Punctation consisting of fine, sparsely distributed punctures.

***Genitalia*:** Median lobe of aedeagus (Fig. 18) sickle-shaped, with evident dorsal and ventral sclerites; dorsal sclerite without surface sculpture and divided into two parts in apical half: left part shorter than right one, both slightly curved, with small crests, notches and truncate apexes (Fig. 18A, B); ventral sclerite divided into two parts apically: left part more strongly sclerotised, broader, shorter, with broadly pointed apex, right part longer, partly sclerotised (medially membranous), with elongate, thin apex in shape of very weak hook (Fig. 18C).

Lateral lobes (parameres) of narrow triangular form, with broader subdistal part due to curved setigerous dorsal margin; setae numerous, dense, and strong distally, and distinctly less numerous, weaker, and sparser basally (Fig. 18D).

**Female.** Identical to male in habitus. Protibia simple, not angled basally and only slightly broadened distally; pro- and mesotarsomeres not broadened, without adhesive setae.

#### Variability.

All specimens of the type series agree well with the holotype. There is only slight variability in body colouration: some specimens have head with pale frontal part and dark vertex, and the sides of elytra are indistinctly paler than elytral disc. Few longitudinal strioles present in depression close to posterior angles of pronotum in one female. Small differences were detected also in the shape of the male median lobe: a notch on right part of dorsal sclerite can be almost absent in some specimens (cf. Figs 18B, 23).

#### Measurements.

TL: 6.3–7.2 mm (mean value: 6.9 ± 0.2 mm); holotype: 6.9 mm. TL-h: 5.7–6.4 mm (mean value: 6.2 ± 0.2 mm); holotype: 6.3 mm. MW: 3.2–3.6 mm (mean value: 3.4 ± 0.1 mm); holotype: 3.4 mm.

#### Differential diagnosis.

Based on the absence of elytral striae, the new species can be included into the *C. hydroporoides* species group (or *haemorrhoidalis*, in earlier studies). This species group contains currently 55 species distributed predominantly in Afrotropical and Neotropical regions (Nilsson and Hájek 2020). Only two species of this group were recently described from the Oriental (Maluku, Indonesia) and Australian regions (Papua New Guinea) by Hájek et al. (2010) and Megna et al. (2017), respectively.

Based on shape of the median lobe and paramere, *C. laevipennis* sp. nov. is without any doubt closely related to *C. variistriatus* sp. nov., which it resembles also in size, body shape, and colouration, and *C. urceolus* sp. nov. The type localities of *C. laevipennis* sp. nov. and *C. variistriatus* sp. nov. are only ca. 20 km apart, although they differ significantly in altitude. Due to great variability of elytral striation in *C. variistriatus* sp. nov. described below, we hesitated first to describe another species, which may represent only a non-striated population of one species. Finally, we have decided to describe both species, because of: 1) *C. laevipennis* sp. nov. has more parallel habitus than *C. variistriatus* sp. nov.; 2) there are no intermediate specimens, i.e., there are no specimens of *C. variistriatus* sp. nov. without elytral striae, and no specimens of *C. laevipennis* sp. nov. with traces of striae; 3) minor differences in the shape of the median lobe can be detected between both species, mainly in shape of the apexes of parts of the dorsal and ventral sclerites (cf. Figs 18, 22–25); 4) there is significant genetic divergence between both species: uncorrected genetic distances between the sequenced CO1 haplotypes of *C. laevipennis* sp. nov. and *C. variistriatus* sp. nov. are ca. 7% (pers. obs.).

#### Etymology.

The species name is composed from Latin adjective *laevis* (-*is*, -*e*, = smooth) and noun *penna* (-*ae*, feminine, = wing), referring to smooth elytra without striae.

#### Distribution.

The species is known only from medium altitude area (ca. 600–900 m) in north-central Guadalcanal.

#### Habitat.

Largely unknown; according to label data, one specimen was collected in a water-filled hole in tree trunk.

### Copelatus portior

Taxon classificationAnimaliaColeopteraDytiscidae

Guignot, 1956

8AF0CA7D-9BD1-5945-BF51-A353D15580A2

Figures 5–6 , 19

Copelatus portior Guignot, 1956: 53 (type locality: “New Hebrides [Vanuatu], Malekula Island”).
Copelatus divisus Watts, 1978: 122 (“Seleo, Berlinhafen” [Papua New Guinea, Sandaun Province, Seleo Island); synonymy by Hendrich et al. (2019).

#### Material examined.

***Guadalcanal*:** 1 ♂, Honiara, M.V. light, 8.–12.ix.1953, J.D. Bradley leg.; 1 ♂, Honiara, Kukum, 1962, P.J.M. Greenslade leg.; 1 ♀, same data, but 19.v.1962; 1 ♂, same data, but 20.v.1963; 1♂, same data, but, 2.iii.1965; 1 ♂, 1 ♀, same data, but 12.v.1966 (all NHMUK); 1 ♂, 1 ♀, Mt. Austine, Barana vill. env., 09°28.0'S, 159°58.4'E, 280 m, 23.xi.-8.xii.2013, J. Hájek leg. (NMPC, ZSMG).

***Ontong Java*:** 1 ♂, 1 ♀, Keila, 31.i.1955, E.S. Brown leg. (NHMUK); 1 ♀, Leuaniua, 27.i.1955, E.S. Brown leg. (NHMUK).

#### Diagnosis.

**Figures 5, 6.**
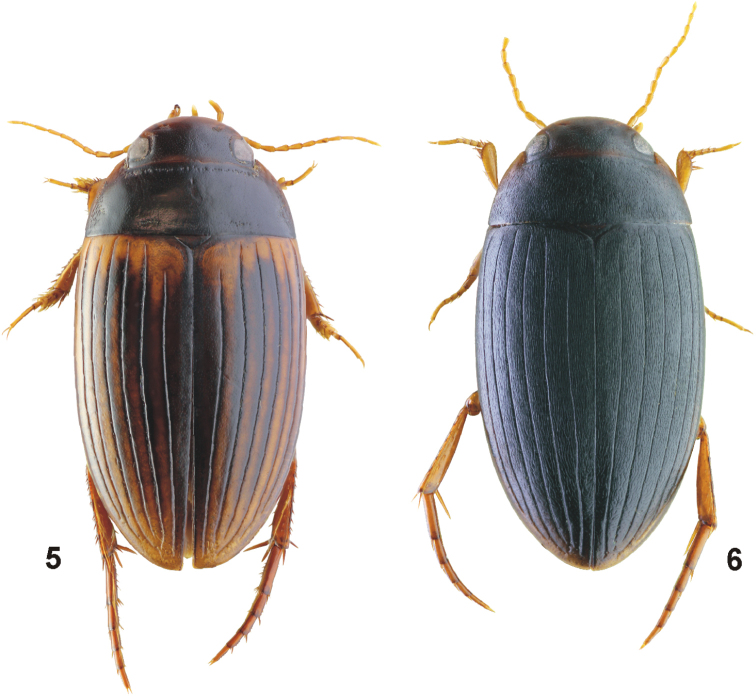
Habitus of *Copelatus***5***C. portior* Guignot, 1956 (male, Guadalcanal; TL: 5.2 mm) **6***C. portior* (striolate female, Papua; TL: 4.7 mm).

For complete description, see Hendrich et al. (2019). Medium sized (TL: 5.0–5.7 mm), oblong-oval species. Species variable in elytral colouration: elytra from almost uniformly dark brown to black with only base and lateral sides yellowish, to almost yellowish-orange coloured with dark stripes along elytral striae (Fig. 5). Elytron with well-impressed six discal striae and a submarginal stria. Female dimorphic; striolate form with dorsal surface almost black, matt and with coarse microreticulation and numerous strioles on elytra and pronotum (Fig. 6). Median lobe in lateral view sickle-shaped; broad and subparallel in basal two thirds, thin and regularly curved in apical third; a distinct hammer-like process present in two thirds on ventral side (Fig. 19). Parameres broad, “D”-shaped; apical lobes moderately long, club-shaped.

#### Distribution.

The species is originally described from northern Papua New Guinea and Vanuatu. It is widely distributed in the Australasian Region: from Lesser Sunda Islands, through New Guinea, northern Australia, and Solomon Islands to Vanuatu. First record from the Solomon Islands.

#### Habitat.

Two specimens from Barana were recently collected in a streamlet flowing through secondary forest and gardens near the village; both of them were found in calm water with decaying leaves on the bottom (Fig. 26).

### Copelatus tulagicus

Taxon classificationAnimaliaColeopteraDytiscidae

Guignot, 1942

224E2F46-8757-58F4-8D27-C066F2D5AFE4

Figures 7 , 8 , 20

Copelatus apicalis J. Balfour-Browne, 1939: 78 (type locality: “Solomon Islands: Tulagi; preoccupied by Copelatus apicalis Fairmaire, 1898: 465 [currently in genus Madaglymbus Shaverdo & Balke, 2008]).
Copelatus tulagicus Guignot, 1942: 86 (as a replacement name for Copelatus apicalis J. Balfour-Browne, 1939: 78).

#### Type material.

***Holotype*:** ♂, labelled: “Type [round label with red frame, p] // 1826 [hw] // SOLOMON IS. [p] / Tulagi / 3.viii.1934 / on leaf. [hw] / R.A.Lever [p] // Pres.by / Imp.Inst.Ent. / B.M.1936-90. [p] // Copelatus / apicalis, / ♂ Type sp. nov. / J.Balfour-Browne [hw]” (NHMUK).

***Paratype*:** ♀, labelled: “Type [round label with red frame, p] // PAPUA: Kokoda [yellow underlined] / I,200ft.v.1933. / L.E.Cheesman. / B.M.1933-577. [p] // Copelatus / apicalis, / ♀ Type sp. nov. / J.Balfour-Browne [hw]” (NHMUK).

#### Additional material examined.

***Guadalcanal*:** 1 ♀, Kukum, 28.iii.1958, E.S. Brown leg.; 1 ♂, 1 ♀, Kukum, 20.v.1963, P. Greenslade leg.; 1 ♀, same data, but 29.v.1963; 3 ♀♀, same data, but 8.i.1965; 1 ♀, same data, but 26.v.1962; 1 ♂, Mt. Austen, xii.1965–i.1966, P. Greenslade leg. (all NHMUK); 1 ♂, ca 4.5 km S of Barana vill., forest near “Japanese camp” at Moka river, 09°30.3'S, 159°58.9'E; 275 m, 5.–6.xii.2013, J. Hájek leg.; 1 ♀, Mt. Austine, Barana vill. env., 09°28.0'S, 159°58.4'E, 280 m, 23.xi.-8.xii.2013, J. Hájek leg. (all NMPC). ***Santa Isabel***: 1 ♀, Ysabel, Gatere, 19.ii.1956, E.S. Brown leg. (NHMUK).

#### Diagnosis.

**Figures 7, 8.**
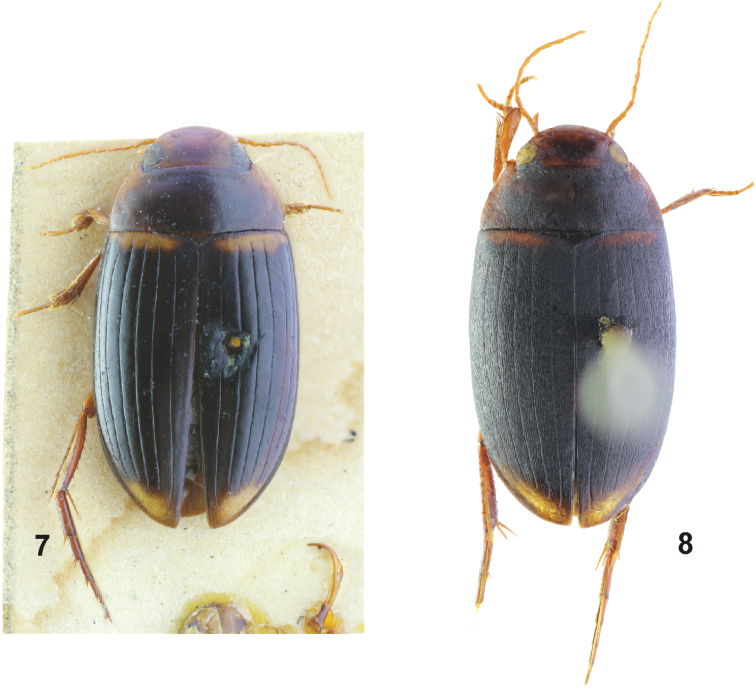
Habitus of *Copelatus***7***C. tulagicus* Guignot, 1942 (holotype; TL: 6.5 mm) **8***C. tulagicus* (striolate female, Guadalcanal; TL: 6.1 mm).

Medium sized (TL: 5.8–6.8 mm), elongate, oblong-oval species. Elytra with transverse testaceous basal band, which does not reach either suture or lateral margin, and with relatively small apical testaceous spot (Figs 7, 8). Pronotum with short longitudinal strioles near posterior angles. Elytra with six well impressed discal striae and a submarginal stria: striae 1 and 5 beginning more posteriorly than other striae; submarginal stria long, beginning at elytral mid length. Female dimorphic; striolate form with dorsal surface matt and with coarse microreticulation and numerous strioles on pronotum and elytra, except for apex (Fig. 8). Median lobe hook-like shaped in lateral view, simple; broadened and with distinct pit in two thirds of its length, apically tapering and strongly curved dorsally (Fig. 20A–C). Parameres “D”-shaped; apical lobes moderately long (Fig. 20D).

#### Comments on classification.

Based on the characteristic hook-like shape of the median lobe, *C. tulagicus* apparently belongs to a complex of species distributed in Sunda Islands and New Guinea, including *C. geniculatus* Sharp, 1882, *C. gentilis* Sharp, 1882, *C. lineatus* (Guérin-Méneville, 1838), *C. biroi* Guignot, 1956, and *C. subterraneus* Guéorguiev, 1978 (of the *C. irinus* species group) and several additional undescribed species; the most closely related species is probably *C. martinbaehri*Hendrich et al., 2019 described recently from southeastern PNG and northern Queensland.

*Copelatus tulagicus* was described based on a male from Solomons and female specimen from southeastern New Guinea (Kokoda). The conspecificity of the female with the male holotype is doubtful with the respect to recently described *C. martinbaehri* from Central Province (Papua New Guinea), which differs from *C. tulagicus* only in the shorter and straighter apical part of the male median lobe of the aedeagus.

#### Distribution.

The species seems to be widely distributed across the New Guinea and the Solomon Islands.

#### Habitat.

The specimen from Barana was recently collected together with *C. portior* in a streamlet flowing through secondary forest and gardens near the village (Fig. 26). The specimen from Moka River was collected in a small puddle near the river. Some specimens from Kukum were covered with moth scales and they were apparently collected at light.

### Copelatus urceolus sp. nov.

Taxon classificationAnimaliaColeopteraDytiscidae

CBB4A4F2-4975-572D-AB8D-E316F2A55391

http://zoobank.org/F1E81914-2D6C-4163-A5E9-6916D097F308

Figures 9 , 21

#### Type locality.

Solomon Islands, Guadalcanal, Vulavula River.

#### Type material.

***Holotype*:** ♂, labelled: “♂ [p] // Type [round label with red frame, p] // SOLOMON IS: / GUADALCANAL / 1720' 6.viii.53. [hw] // Small pool on top / of large boulder in Vulavula River. [hw] // Brit.Mus. / 1987-14 [p] // aD 2 [hw] // Copelatus / urceolus Type! [hw] / J. Balfour-Browne det., 195 [p] 3 [hw]” (NHMUK).

#### Description of male holotype.

**Figures 9–12.**
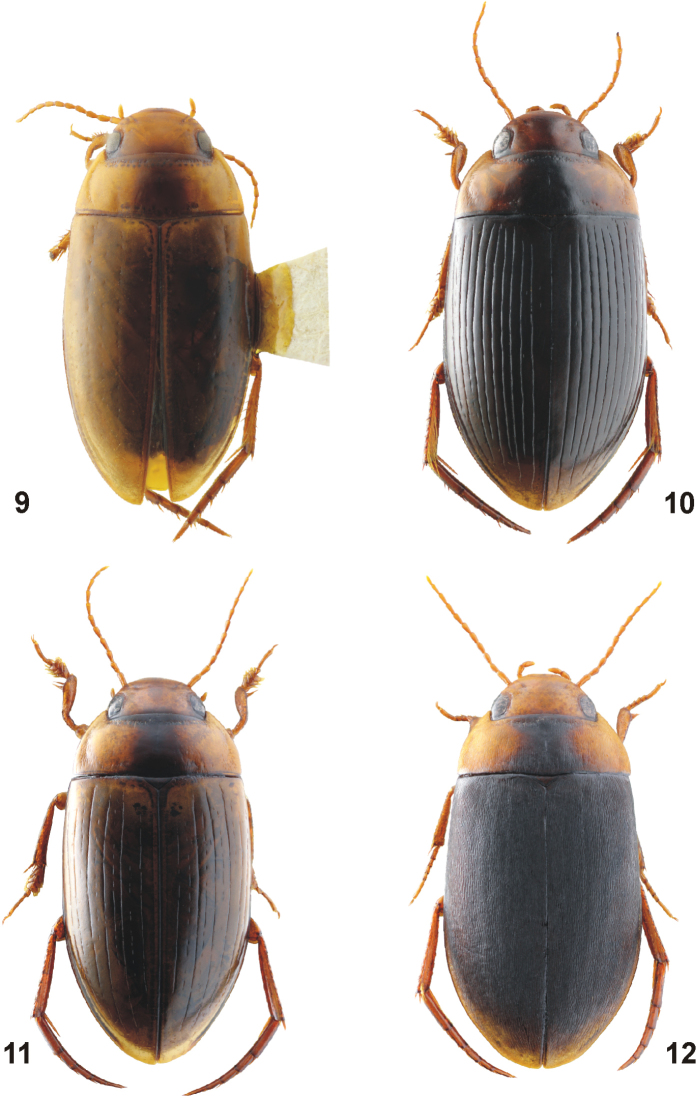
Habitus of *Copelatus***9***C. urceolus* sp. nov. (holotype, TL: 5.1 mm) **10***C. variistriatus* sp. nov. (holotype; TL: 6.6 mm) **11***C. variistriatus* sp. nov. (male paratype with reduced striae; TL: 6.2 mm) **12***C. variistriatus* sp. nov. (striolate female paratype; TL: 5.9 mm).

***Habitus*:** Elongate, oblong-oval, broadest before mid-length of elytra; body distinctly convex in lateral view. Body outline continuous, without discontinuity between pronotum and elytra. Dorsal surface shiny (Fig. 9).

***Colouration*:** Head, lateral parts of pronotum, appendages and prosternum orange-ferruginous; elytra, meso- and metaventrite, and abdomen pitchy brown; anterior margin and midpart of pronotum darkest, brown.

***Head*:** Moderately broad, ca. 0.60 × width of pronotum, trapezoidal. Anterior margin of clypeus indistinctly concave. Antenna with antennomeres long and slender. Reticulation consisting of moderately deeply impressed isodiametric meshes. Punctation double, consisting of coarse setigerous punctures, and very small punctures spread sparsely on surface; row of coarse punctures present around inner margin of eyes, few punctures present at frontal level of eyes, and several punctures anterolaterally to eyes in fronto-clypeal depressions.

***Pronotum*:** Transverse (width/length ratio = 2.48), broadest between posterior angles, lateral margins moderately curved. Sides with lateral beading thin, but distinct except for anterior angles. Reticulation similar to that of head. Punctation similar to that of head; rows of coarse setigerous punctures present along anterior margin, laterally in longitudinal depression close to sides, several punctures present also in basolateral depressions along basal margin. Centre of disc with medial longitudinal smooth line.

***Elytra*:** Base of elytra as broad as pronotal base; lateral margins of elytra curved, distinctly narrowing in apical half. Elytral striae absent. Reticulation similar to that of head and pronotum. Punctation consisting of coarse setigerous punctures and very fine sparse punctures. Coarse punctures arranged in three distinct longitudinal puncture lines: two discal and lateral; another row of punctures present along lateral margin of elytra, and few coarse punctures present also in interspace between discal and lateral puncture lines.

***Legs*:** Protibia modified, angled near base, distinctly broadened anteriorly, club shaped. Pro- and mesotarsomeres 1–3 distinctly broadened, with adhesive setae on their ventral side.

***Ventral side*:** Prosternum sinuate anteriorly, obtusely keeled medially. Prosternal process shortly lanceolate, in cross-section convex, apex obtuse; process distinctly bordered; reticulation almost effaced. Metaventrite with microsculpture consisting of polygonal meshes; lateral parts of metaventrite (“metasternal wings”) tongue-shaped, slender. Metacoxal lines nearly complete, absent only very close to metaventrite. Metacoxal plates covered laterally with long, deep longitudinal strioles; reticulation consisting of extremely elongated, longitudinal polygonal meshes. Metacoxal processes rounded and incised at posterior margin. Abdominal ventrites I–II with longitudinal strioles. Tuft of setae present antero-medially on ventrites III–V; ventrite VI with setigerous punctures laterally on either side. Abdominal reticulation consisting of elongate polygonal meshes, longitudinal on ventrites I and II, oblique on ventrite III, and transverse on ventrites IV–VI. Punctation consisting of fine, sparsely distributed punctures.

***Genitalia*:** Median lobe of aedeagus (Fig. 21) sickle-shaped, with evident dorsal and ventral sclerites; dorsal sclerite without surface sculpture and divided into two parts in apical half: left part distinctly shorter than right one, parts slightly curved, with crests and broadly pointed apexes (Fig. 21A, B); ventral sclerite divided into two parts apically: left part sclerotised, broader, shorter, with broadly pointed apex, right part longer, partly sclerotised (apically membranous), asymmetrically concave, with long, thin, rounded apex (Fig. 21C).

Lateral lobes (parameres) of narrow triangular form, with broader subdistal part due to curved setigerous dorsal margin; setae numerous, dense, and strong distally, and distinctly less numerous, weaker, and sparser basally (Fig. 21D).

**Female.** Unknown.

#### Measurements.

TL: 5.1 mm. TL-h: 4.5 mm. MW: 2.5 mm.

#### Differential diagnosis.

Member of the *Copelatus hydroporoides* species group, see under *C. laevipennis* sp. nov. *Copelatus urceolus* sp. nov. is most likely related to *C. laevipennis* sp. nov. and *C. variistriatus* sp. nov. It differs from both mentioned species in smaller body length, different shape of median lobe of male genitalia (cf. Figs 19, 21, 22), and from the latter species also in absence of elytral striae.

#### Etymology.

We adopted the manuscript name used by J. Balfour-Browne. Latin noun *urceolus* (-*i*, masculinum) means small pitcher or jug, referring probably to the habitat in which the type specimen was caught; the name is used in the nominative case, standing in apposition.

#### Distribution.

The species is known only from the type locality in central Guadalcanal.

### Copelatus variistriatus sp. nov.

Taxon classificationAnimaliaColeopteraDytiscidae

170C4900-CA51-5767-8983-471B290F03EA

http://zoobank.org/A5542699-AEEA-4B6F-A5F2-629054351795

Figures 10–12 , 22 , 24 , 25

#### Type locality.

Solomon Islands, Guadalcanal, 4.5 km S of Barana Village, Moka River near “Japanese camp”, 09°30.3'S, 159°58.9'E.

#### Type material.

***Holotype*:** ♂, labelled: “Solomon Islands, GUADALCANAL / ca 4.5 km S of Barana vill., forest / nr. “Japanese camp” & Moka river / 09°30.3'S, 159°58.9'E; 275 m / Jiří Hájek leg., 5.–6.xii.2013 [printed] // HOLOTYPE ♂ / *COPELATUS* / *variistriatus* sp. nov. / Hájek, Hendrich & Balke det. 2018 [red label, printed]” (NMPC). ***Paratypes***: 9 ♂♂, 5 ♀♀, same data as holotype (NHMUK, NMPC); 9 ♂♂, 11 ♀♀, labelled: “Solomon Islands, GUADALCANAL / ca. 3.5 km SE of Barana vill. / (drying up stream in shaded gorge) / 09°29.8'S, 159°59.5'E; 190 m / Jiří Hájek leg., 24.xi.-14.xii.2013 [printed]” (NHMW, NMPC, ZSMG). All paratypes with the respective printed red label.

#### Description of male holotype.

***Habitus*:** elongate oblong oval, broadest at mid-length of elytra; body distinctly convex in lateral view. Body outline continuous, without discontinuity between pronotum and elytra. Dorsal surface shiny (Fig. 10).

***Colouration*:** Body colour pitchy brown; head, sides of pronotum and appendages paler, ferruginous.

***Head*:** Rather narrow, ca. 0.57 × width of pronotum, trapezoidal. Anterior margin of clypeus indistinctly concave. Antenna with antennomeres long and slender. Reticulation consisting of moderately deeply impressed isodiametric meshes. Punctation double, consisting of coarse setigerous punctures, and very small punctures spread sparsely on surface; row of coarse punctures present around inner margin of eyes, few punctures present at frontal level of eyes, and several punctures anterolaterally to eyes in fronto-clypeal depressions.

***Pronotum*:** Transverse, broadest between posterior angles (width/length ratio = 2.91), lateral margins moderately curved. Sides with lateral beading very thin and indistinct. Reticulation similar to that of head. Punctation similar to that of head; rows of coarse setigerous punctures present along anterior margin, laterally in longitudinal depression close to sides, several punctures present also in basolateral depressions along basal margin. Few longitudinal strioles present in depressions close to posterior angles; disc of pronotum with shallow medial longitudinal scratch.

***Elytra*:** Base of elytra as broad as pronotal base; lateral margins of elytra slightly diverging in basal half, distinctly narrowing in apical half. Eleven discal (but see variability) and a submarginal longitudinal striae present on each elytron: distance between stria 1 and suture twice bigger than distance between other discal striae; striae 1 and 2 absent at base; stria 10 present only as numerous strioles in basal third of elytra; even striae shortened apically. Submarginal stria rather short, present approximately in third fourth of elytral length. Few longitudinal strioles present in interspaces between suture, stria 1, and stria 2. Reticulation similar to that of head and pronotum, meshes somewhat elongated longitudinally. Punctation consisting of coarse setigerous punctures and very fine sparse punctures; coarse punctures present in row along lateral margin of elytra.

***Legs*:** Protibia modified, angled near base, distinctly broadened anteriorly, club shaped. Pro- and mesotarsomeres 1–3 distinctly broadened, with adhesive setae on their ventral side.

***Ventral side*:** Prosternum sinuate anteriorly, obtusely keeled medially. Prosternal process shortly lanceolate, in cross-section convex, apex obtuse; process distinctly bordered laterally; reticulation consisting of shallow, hardly perceptible polygonal meshes. Metaventrite with microsculpture consisting of polygonal meshes; lateral parts of metaventrite (“metasternal wings”) tongue-shaped, slender. Metacoxal lines nearly complete, absent only very close to metaventrite. Metacoxal plates covered with long, deep longitudinal strioles; reticulation consisting of extremely elongated, longitudinal polygonal meshes. Metacoxal processes rounded at posterior margin. Abdominal ventrites I–II with longitudinal strioles; ventrites III and IV with oblique strioles laterally. Tuft of setae present antero-medially on ventrites III–V; ventrite VI with setigerous punctures laterally on either side. Abdominal reticulation consisting of elongate polygonal meshes, longitudinal on ventrites I and II, oblique on ventrite III and transverse on ventrites IV–VI. Punctation consisting of fine, sparsely distributed punctures.

***Genitalia*:** Median lobe of aedeagus (Fig. 22) sickle-shaped, with evident dorsal and ventral sclerites; dorsal sclerite without surface sculpture and divided into two parts in apical half: left part shorter than right one, both slightly curved, with small crests, notches and truncate apexes (Fig. 22A, B); ventral sclerite divided into two parts apically: left part more strongly sclerotised, broader, shorter, with broadly pointed apex, right part longer, partly sclerotised (medially membranous), with elongate, thin apex in shape of weak hook (Fig. 22C).

Lateral lobes (parameres) of narrow triangular form, with broader subdistal part due to curved setigerous dorsal margin; setae numerous, dense, and strong distally, and distinctly less numerous, weaker and sparser basally (Fig. 22D).

**Female.** Similar to male in habitus. Protibia simple, not angled basally and only slightly broadened distally; pro- and mesotarsomeres not broadened, without adhesive setae. Dimorphic; striolate form due to dense striolation matt; strioles present on whole surface of pronotum and elytra, thus elytral striation not recognisable: strioles longitudinal, usually very long, only rarely confluent (Fig. 12).

#### Variability.

The specimens of the type series vary in dorsal body colouration: specimens from drying up stream are generally paler (ochreous) than specimens from Moka River. The highest variability is however in elytral striation: in some specimens short strioles present between suture and stria 1, suggesting the present of “true” stria 1, which is vanished in all studied specimens (thus, the first visible stria is actually stria 2); in addition, strioles presenting in basal third between striae 9 and 10 may confluent into distinct stria in some specimens. On the other hand, all even striae have tendency for reduction, they are often fragmented, persisting only as a few longitudinal strioles or missing completely; in the extreme, only four elytral striae (stria 1, 3, 5, 7) are well preserved on disc with striae 9 and 10 preserved as short strioles (Fig. 11). The submarginal stria is missing in ca. one third of all specimens. Generally, the striation is more complete in the specimens from Moka River than in specimens from drying up stream. Additional short strioles may occur irregularly between all discal striae in some specimens. Small differences were detected also in the shape of the male median lobe (cf. Figs 22B, 24, 25).

#### Measurements.

TL: 5.7–6.7 mm (mean value: 6.4 ± 0.2 mm); holotype: 6.6 mm. TL-h: 5.2–6.1 mm (mean value: 5.7 ± 0.2 mm); holotype: 6.0 mm. MW: 2.9–3.4 mm (mean value: 3.2 ± 0.1 mm); holotype: 3.3 mm.

#### Differential diagnosis.

Due to variable number of elytral striae, it is quite difficult to classify the new species within the traditional *Copelatus* species group (Sharp 1882; Guignot 1961; Guéorguiev 1968). We have tentatively included the species into the *C. trilobatus* species group, as the state of eleven dorsal striae and a submarginal stria is the most frequent condition in *C. variistriatus* sp. nov., and the other forms resulting from subsequent reduction of striae. However, the double distance between suture and stria 1 suggested possible presence of twelfth stria in the ground plan of the species, currently absent in all specimens studied.

The *C. trilobatus* species group includes up to now 24 species occurring in tropics of all continents (see under *C. bougainvillensis* sp. nov.). However, no species is similar to the new species. *Copelatus variistriatus* sp. nov. is without any doubts closely related to *C. laevipennis* sp. nov., from which it differs in slightly smaller body length, more oval habitus, presence of elytral striae, and minor differences in the shape of the median lobe (see also under *C. laevipennis* sp. nov.).

#### Etymology.

The species name is composed from Latin adjectives *varius* (-*a*, -*um*, = diverse, variegated) and *striatus* (-*a*, -*um*, = with striae), referring to the variable number of elytral striae in the new species.

#### Distribution.

The species is known so far only from two localities, ca. 1.5 km apart, along north coast of Guadalcanal.

#### Habitat.

At the type locality, the species was collected in small side rock pools of a small forest river. At the other locality, the specimens were collected in puddles/pools with muddy bottom made by a temporary forest stream (Fig. 28). At both places it was collected together with *C. baranensis* sp. nov.

### Copelatus

Taxon classificationAnimaliaColeopteraDytiscidae

sp. 1

6AF30C61-9990-5005-8FF9-8FE667C6E60D

Figure 13

#### Material examined.

***Guadalcanal*:** 1 ♀, 0.5 km N Mbaole, 09°37.69'S, 160°06.69'E, 2799 feet, 2007, K. Mailautoka leg. (ZSMG).

#### Diagnosis.

**Figures 13, 14.**
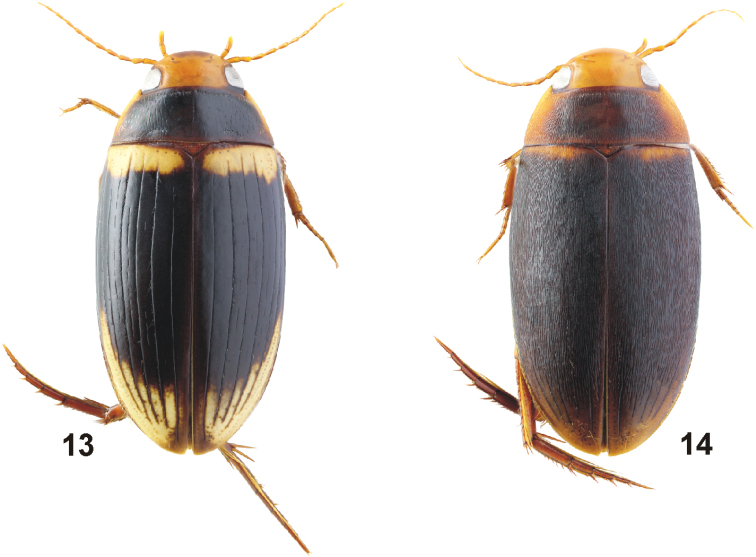
Habitus of *Copelatus***13***Copelatus* sp. 1 **14***Copelatus* sp. 2.

Medium sized (TL: 6.3 mm), elongate, oblong-oval species. Head testaceous, with dark band posterior to eyes; pronotum brown blackish, with testaceous sides; elytra brown blackish, with broad transverse testaceous basal band and testaceous apical part. Pronotum with short longitudinal strioles laterally. Elytra with ten discal striae and a submarginal stria: striae 1, 3, 5, 7, 9, and 10 almost complete and well impressed; striae 2, 4, 6, and 8 present only as a series of short strioles between odd striae; submarginal stria short, split to several strioles on one side (Fig. 13).

#### Comments to classification.

Based on presence of ten discal and a submarginal stria on elytra, the species can be included in the *C. erichsonii* species group. It could not be associated with any species currently known from the Solomon Islands. Without a male available for the study, we leave this taxon unidentified to species level.

#### Distribution.

The species is known only from a single medium altitude locality in north-central Guadalcanal.

### Copelatus

Taxon classificationAnimaliaColeopteraDytiscidae

sp. 2

9D703EEA-8536-55C4-9CD2-2DE5836130A8

Figure 14

#### Material examined.

***Guadalcanal*:** 3 ♀♀, 0.5 km N Mbaole, 09°37.69'S, 160°06.69'E, 2799 feet, 2007, K. Mailautoka leg. (ZSMG).

#### Diagnosis.

Medium sized (TL: 7.2–7.9 mm), elongate, oblong-oval species. Head testaceous, with dark band posterior to eyes; pronotum brownish, with broad testaceous sides; elytra brown blackish, with thin transverse testaceous basal band and somewhat paler brown apical part. Pronotum and elytra (except for apical fifth) densely covered with long longitudinal strioles. Elytral striation due to presence of striolae could not be observed; presence of at least five discal striae is perceptible in the non-striolate apical part of elytra (Fig. 14).

#### Comments to classification.

The striolate female could not be associated with any current species group of *Copelatus*. They do not fit to any currently known species from the Solomon Islands. Without a male available for study, we leave this taxon unidentified to species level.

#### Distribution.

The species is known only from a single medium altitude locality in north-central Guadalcanal.

**Figure 15.**
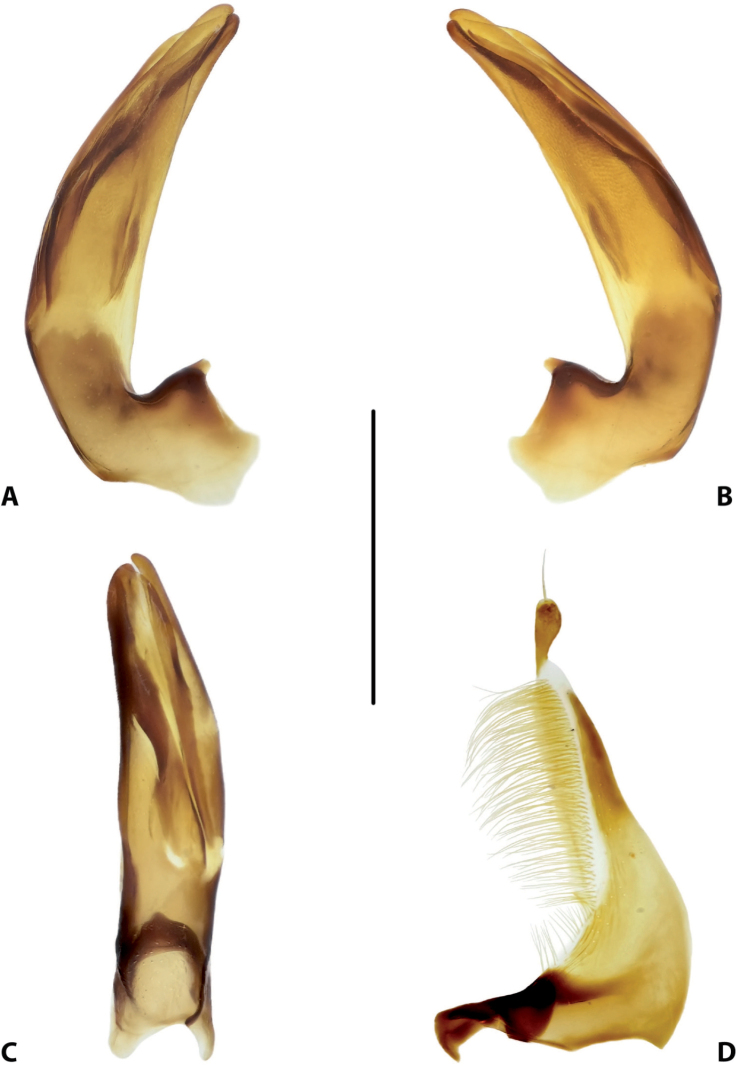
Male genitalia of *Copelatus baranensis* sp. nov. (holotype) **A** median lobe in lateral view, right side **B** median lobe in lateral view, left side **C** median lobe in ventral view **D** left paramere in external view. Scale bar: 0.5 mm.

**Figure 16.**
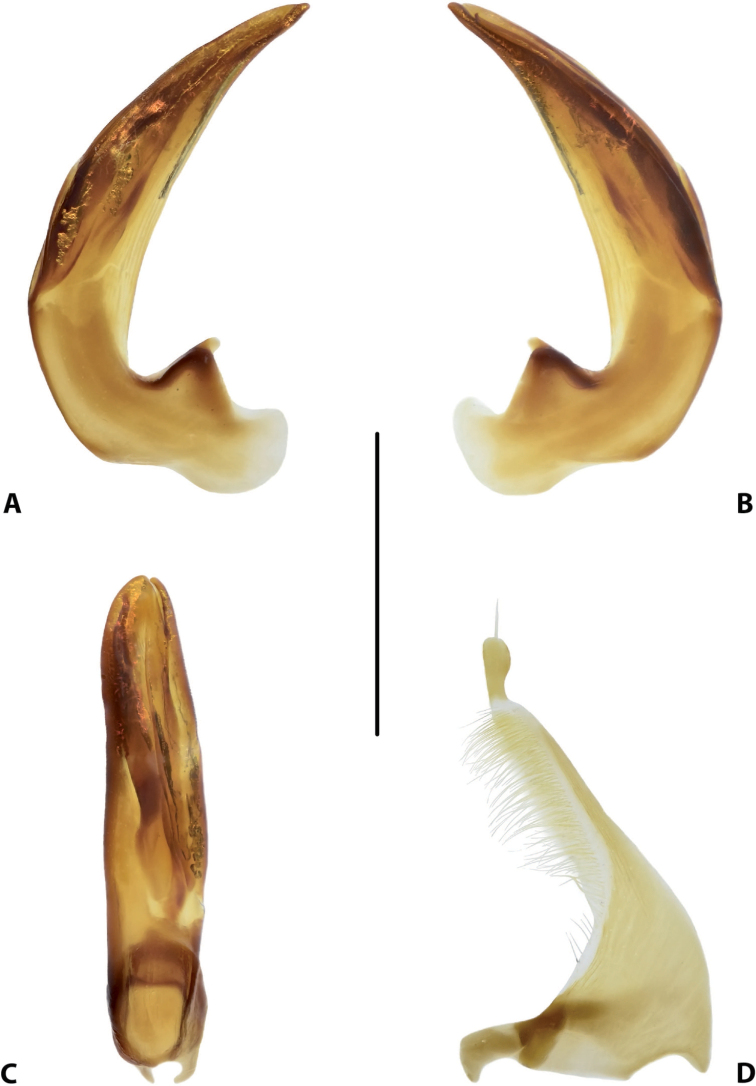
Male genitalia of *Copelatus bougainvillensis* sp. nov. (holotype) **A** median lobe in lateral view, right side **B** median lobe in lateral view, left side **C** median lobe in ventral view **D** left paramere in external view. Scale bar: 0.5 mm.

**Figure 17.**
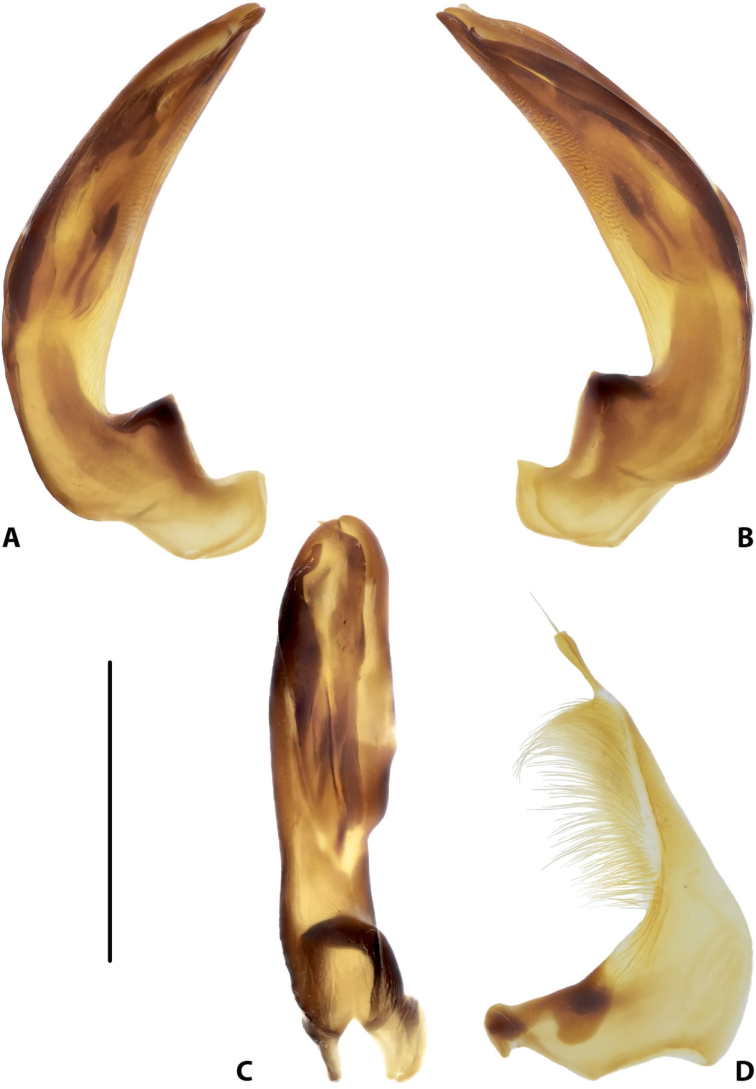
Male genitalia of *Copelatus kietensis* sp. nov. (holotype) **A** median lobe in lateral view, right side **B** median lobe in lateral view, left side **C** median lobe in ventral view **D** left paramere in external view. Scale bar: 0.5 mm.

**Figure 18.**
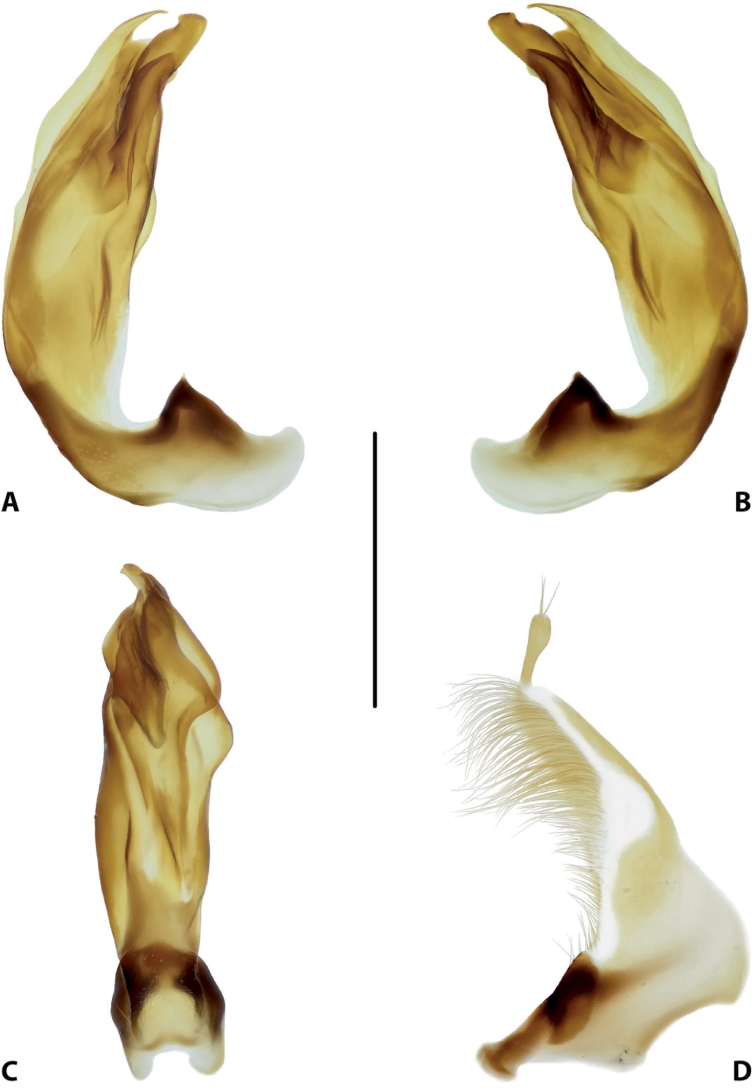
Male genitalia of *Copelatus laevipennis* sp. nov. (holotype) **A** median lobe in lateral view, right side **B** median lobe in lateral view, left side **C** median lobe in ventral view **D** left paramere in external view. Scale bar: 0.5 mm.

**Figure 19.**
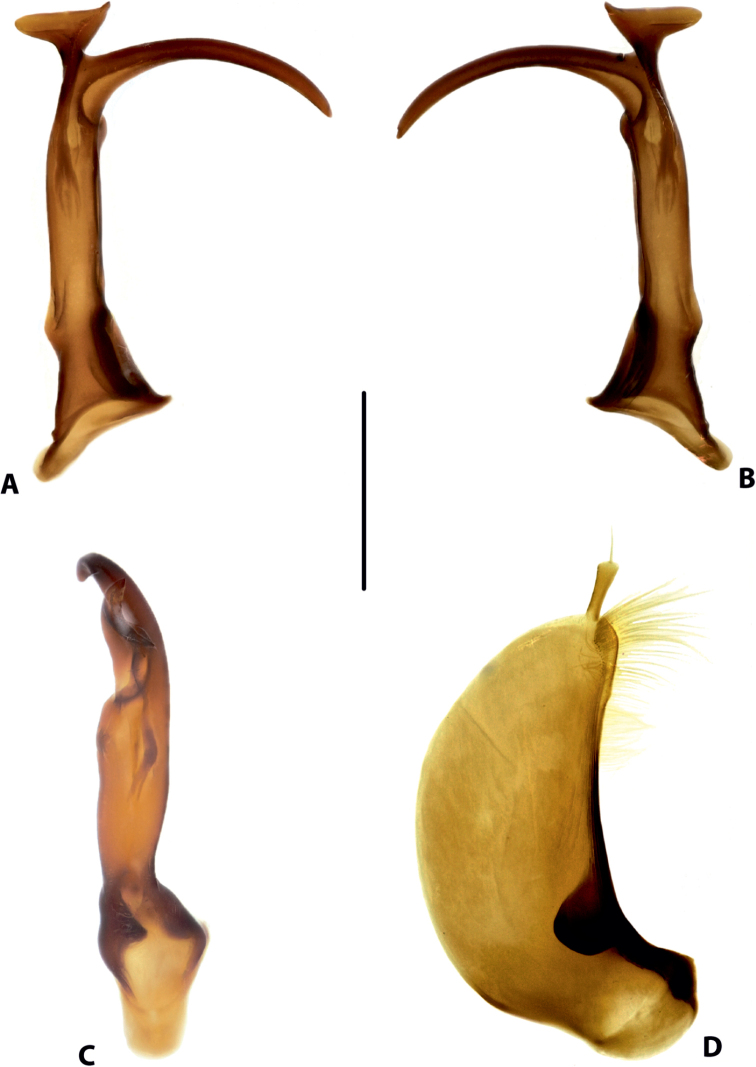
Male genitalia of *Copelatus portior* Guignot, 1956 (Australia) **A** median lobe in lateral view, right side **B** median lobe in lateral view, left side **C** median lobe in ventral view **D** left paramere in external view. Scale bar: 0.5 mm.

**Figure 20.**
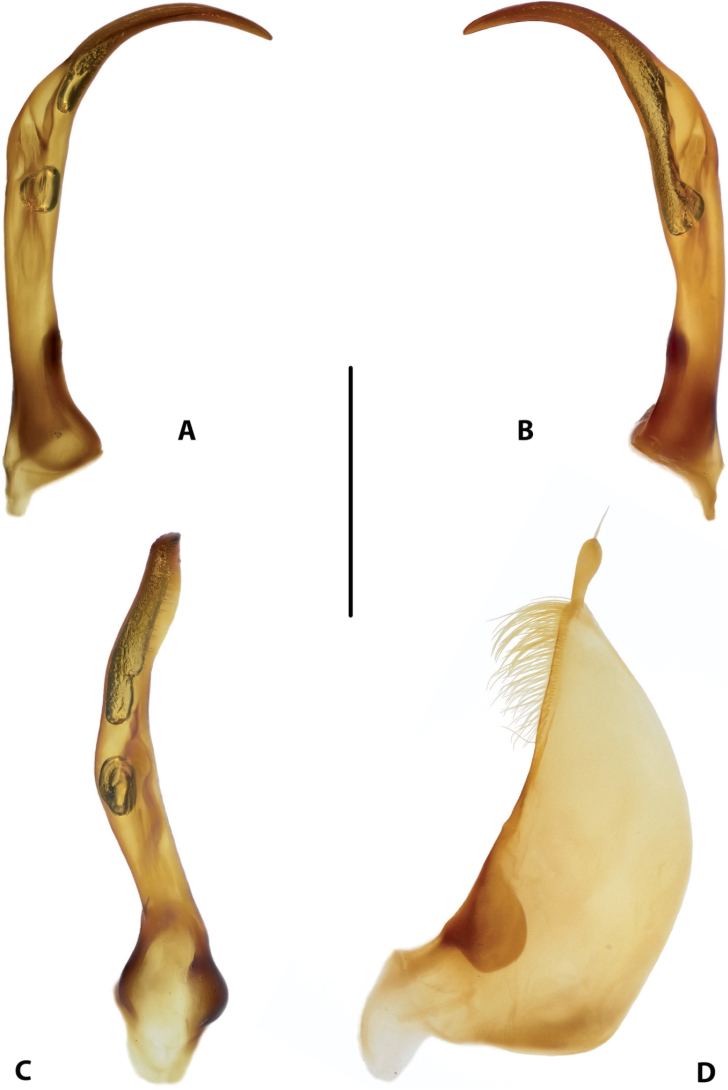
Male genitalia of *Copelatus tulagicus* Guignot, 1942 (holotype) **A** median lobe in lateral view, right side **B** median lobe in lateral view, left side **C** median lobe in ventral view **D** left paramere in external view. Scale bar: 0.5 mm.

**Figure 21.**
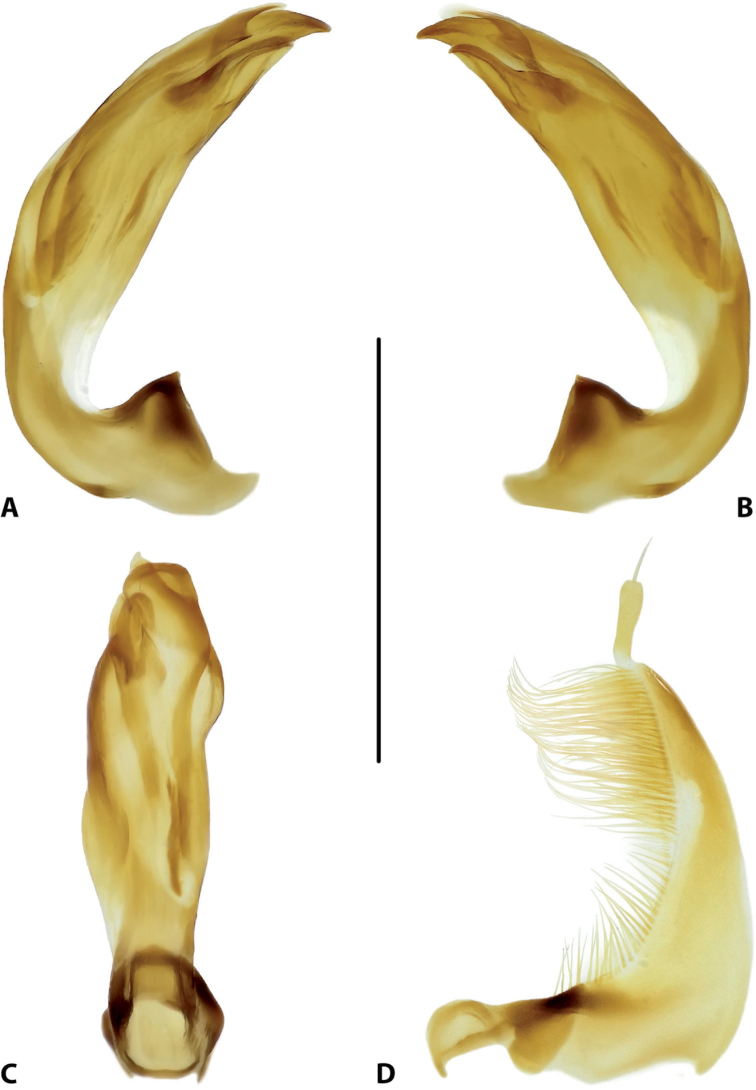
Male genitalia of *Copelatus urceolus* sp. nov. (holotype) **A** median lobe in lateral view, right side **B** median lobe in lateral view, left side **C** median lobe in ventral view **D** left paramere in external view. Scale bar: 0.5 mm.

**Figure 22.**
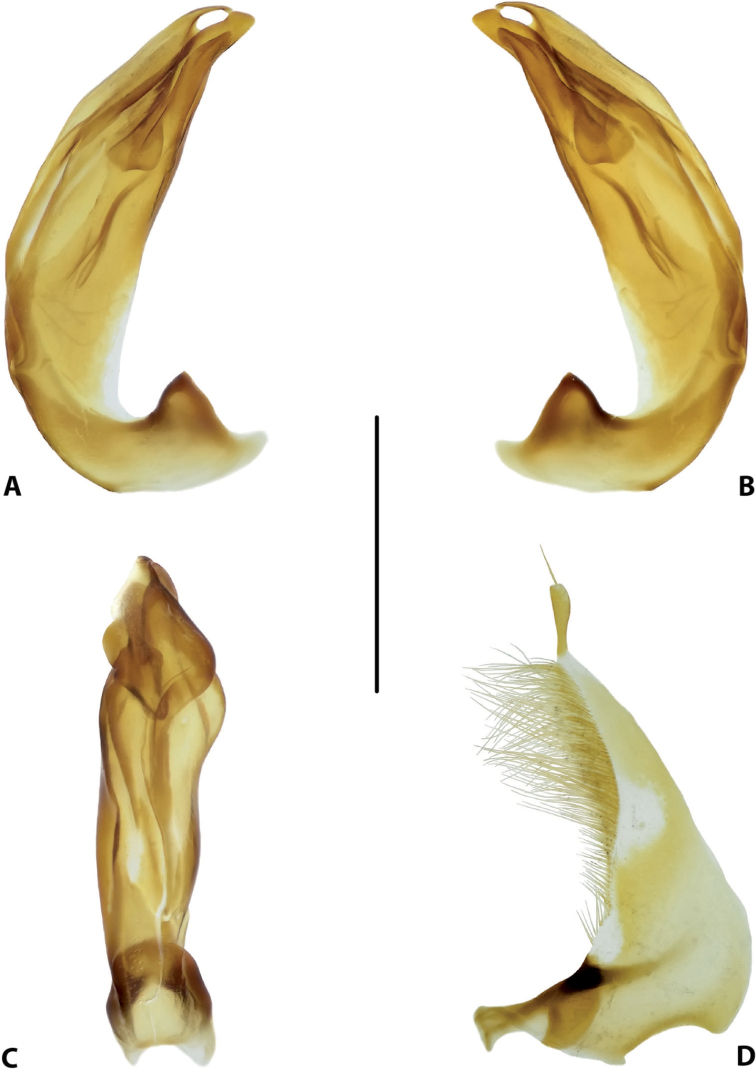
Male genitalia of *Copelatus variistriatus* sp. nov. (holotype) **A** median lobe in lateral view, right side **B** median lobe in lateral view, left side **C** median lobe in ventral view **D** left paramere in external view. Scale bar: 0.5 mm.

**Figures 23–25.**
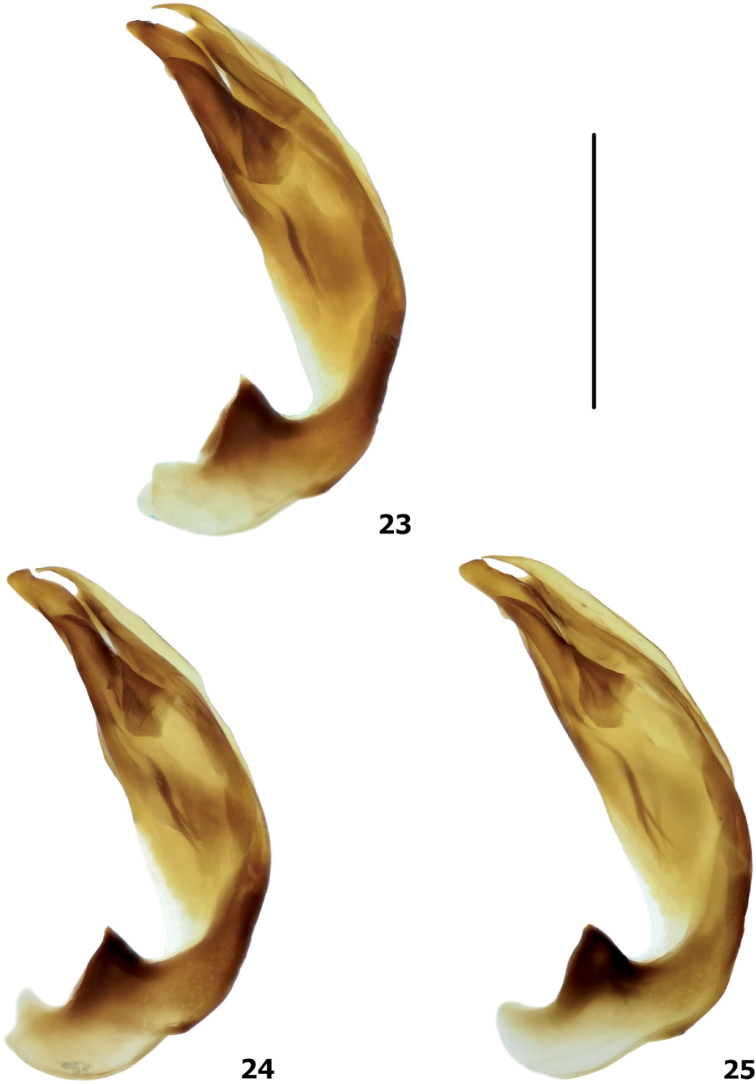
Variability of *Copelatus* male genitalia: median lobe in lateral view, left side **23***Copelatus laevipennis* sp. nov. (paratype) **24, 25***C. variistriatus* sp. nov. (paratypes). Scale bar: 0.5 mm.

**Figures 26–28.**
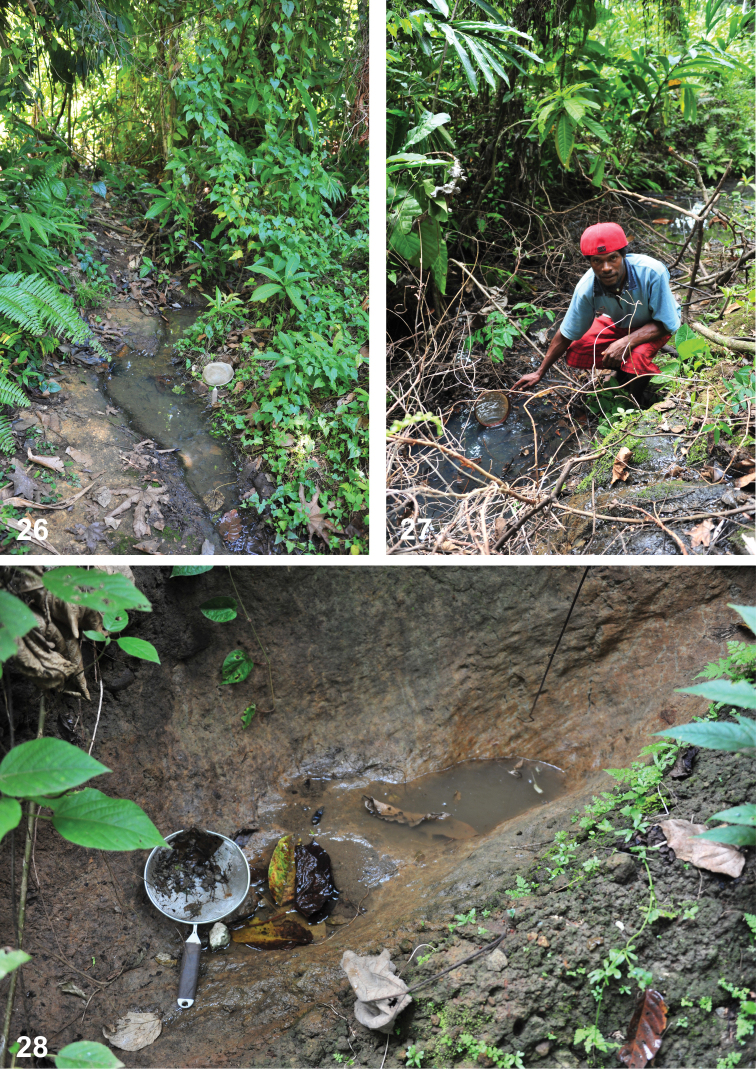
Habitats of *Copelatus* in Guadalcanal**26** temporary forest stream near Barana village **27** shaded pool on the same stream **28** small temporary puddles in a gorge along the road from Barana to Lungga river.

### Key to *Copelatus* species of Solomon Islands

The key is based mostly on male characters, since some species are similar in external morphology and in most cases, the male genitalia need to be studied for reliable species identification. The two unidentified species are not included in the key.

**Table:** 

1	Elytron with six discal striae and a submarginal stria (6+1). Median lobe of aedeagus simple, without division into dorsal and ventral sclerites, sometimes with a median process on ventral side	**2**
–	Elytron with number of striae and degree of their development very variable among species and within one species (0–11 + 0–1). Median lobe of aedeagus complex, with evident dorsal and ventral sclerites, which apically divided into two parts of different shape	**3**
2	Smaller (TL: 5.0–5.7 mm), broader species (Figs 5, 6). Median lobe of aedeagus with a median, hammer-like process on ventral side (Fig. 19)	*** portior ***
–	Larger (TL: 5.8–6.8 mm), more elongate species (Figs 7, 8). Median lobe of aedeagus simple, hook-like, without process (Fig. 20)	*** tulagicus ***
3	Elytron with striae. Dorsal sclerite of median lobe of aedeagus with rugose surface sculpture distinctly visible in lateral view; apexes of two parts of dorsal and ventral sclerites without strong modification, elongate, more or less pressed together (Figs 15–17)	**4**
–	Elytron with or without striae. Dorsal sclerite of median lobe of aedeagus without surface sculpture, smooth; apexes of two parts of dorsal and ventral sclerites differently modified, usually disposed more freely (Figs 18, 20, 21)	**6**
4	Larger species (TL: 6.3 mm). Elytron black with large subapical testaceous spot; with 11 dorsal and a submarginal stria (Fig. 3). Male genitalia as in Fig. 17	***kietensis* sp. nov.**
–	Smaller species (TL: 5.2–6.2 mm). Elytron brownish black with testaceous transverse basal band, or almost uniformly ferruginous; with 11 dorsal striae and with or without submarginal stria (Figs 1, 2)	**5**
5	More parallel species; disc of elytra brownish black (Fig. 1). Median lobe of aedeagus and paramere broader, with more rounded apexes (Fig. 15)	***baranensis* sp. nov.**
–	More oval species; disc of elytra ferruginous, darker along striae (Fig. 2). Median lobe of aedeagus and paramere more elongate and slender apically (Fig. 16)	***bougainvillensis* sp. nov.**
6	Elytra without striae	**7**
–	Each elytron with 11 dorsal and a submarginal stria. The striae can be reduced or additional strioles can be present (Figs 10–12). Male genitalia as in Fig. 22	***variistriatus* sp. nov.**
7	Smaller (TL: 5.1 mm), more oval species (Fig. 9). Male genitalia as in Fig. 21	***urceolus* sp. nov.**
–	Larger (TL: 6.3–7.2 mm), more parallel species (Fig. 4). Male genitalia as in Fig. 18	***laevipennis* sp. nov.**

## Discussion

With ten recorded species, our account of *Copelatus* from the Solomon Islands has to be considered very preliminary. The only island with extensive collecting efforts was Guadalcanal, from where we report eight species, four of which are described as new, two species are widespread in the Australian region, and two species known only from females remain unidentified. However, still only a small (north-central) area of that island was explored and covers only low and medium altitudes. Based on decades of our fieldwork experience in other parts of the world, we suggest that the diversity of *Copelatus* changes altitudinally with different microhabitats. That in turn means that the actual number of *Copelatus* species in Guadalcanal might be at least twice as high. Interestingly, the Copelatinae genus *Exocelina* Broun, 1886, highly diverse in Australia, New Guinea, and New Caledonia (and single species in Vanuatu and Hawaii), has not been reported from the Solomon Islands yet. Most species of *Exocelina* inhabit stream associated stagnant water habitats such as pools in intermittent streams, small waterfilled holes on rocks, and small areas stagnant water at the margin of streams and even the tiniest forest creeks. They can be found in wet gravel and leaves in otherwise dry creek beds. Their apparent absence in the Solomon Islands suggests that this habitat type could be filled by *Copelatus* species (see Toussaint et al. 2015).

## Supplementary Material

XML Treatment for Copelatus baranensis

XML Treatment for Copelatus bougainvillensis

XML Treatment for Copelatus kietensis

XML Treatment for Copelatus laevipennis

XML Treatment for Copelatus portior

XML Treatment for Copelatus tulagicus

XML Treatment for Copelatus urceolus

XML Treatment for Copelatus variistriatus

XML Treatment for Copelatus

XML Treatment for Copelatus
